# Factors Related to the Etiology of Hallux Abducto Valgus: A Systematic Review

**DOI:** 10.3390/jfmk11010117

**Published:** 2026-03-13

**Authors:** Marta María Moreno-Fresco, Stephen Mizzi, Pedro V. Munuera-Martínez, Priscila Távara-Vidalón

**Affiliations:** 1 Department of Podiatry, University of Seville, 41009 Seville, Spain; martamorenof19@hotmail.com (M.M.M.-F.); stavara@us.es (P.T.-V.); 2 Department of Podiatry, University of Malta, Msida MSD2080, Malta

**Keywords:** hallux valgus, etiology, inheritance, foot, first ray

## Abstract

**Background:** The origin of hallux abducto valgus (HAV) is considered to be multifactorial; however, evidence regarding the factors involved in its development is scattered and often contradictory. Understanding the factors that contribute to the onset of HAV is crucial for informing both prevention and clinical management strategies. This review aims to explore the etiological factors associated with the development of HAV. **Methods**: A literature search was conducted in PubMed, Embase, Web of Science and Scopus. The search included observational studies that investigated etiological or risk factors related to the development of HAV. Methodological quality was assessed using the Joanna Briggs Institute (JBI) checklists, and the level of evidence was classified according to the Oxford Centre for Evidence-Based Medicine (OCEBM). **Results**: A total of 36 observational studies (20 cross-sectional and 16 case–control) were included, involving 14,500 participants, predominantly females. Genetic evidence indicated strong familial aggregation and variants in collagen- and extracellular matrix-related genes as potential hereditary determinants. The most consistent biomechanical factors were first-ray hypermobility, abnormal foot pronation and reduced activity of the abductor hallucis muscle. Additionally, female sex, older age and prolonged use of narrow or inadequate footwear were identified as recurring predictive variables. Overall, the findings support a complex etiological model based on the interaction of intrinsic and extrinsic factors. **Conclusions:** The development of HAV appears to be determined by the interaction of genetic, structural and biomechanical factors that alter first-ray stability and forefoot function. Current evidence supports a multifactorial etiological model with a strong hereditary component and higher susceptibility in women. Longitudinal studies employing standardized methods are needed to establish causal relationships and quantify the relative contribution of each factor.

## 1. Introduction

Hallux abducto valgus (HAV) is a foot deformity affecting the first ray, characterized by subluxation of the first metatarsophalangeal joint (1st MTPJ), accompanied by lateral deviation of the hallux and, in severe cases, plantarflexion and eversion of the great toe. The deformity also involves medial deviation of the first metatarsal with dorsiflexion and inversion, often associated with a medial exostosis at the head of the first metatarsal, commonly referred to as a bunion [1,2].

The prevalence of HAV increases with age, affecting approximately 23% of adults between 18 and 65 years and 36% of individuals over 65 years [3]. Recent epidemiological studies continue to confirm its high global prevalence, estimated at approximately 19% in the general population, with higher rates observed among women and older individuals [4]. Significant sex-related differences have also been reported, with women being 2.3 times more likely to develop HAV than men, with prevalence rates of 30% and 13%, respectively [3]. Despite its high prevalence, the exact etiology of HAV remains unclear. It is widely recognized as a multifactorial condition influenced by both intrinsic and extrinsic factors that contribute to the development and progression of the deformity [1,5].

HAV can lead to functional impairments, biomechanical alterations, and substantial pain, negatively impacting patients’ quality of life. Over the years, numerous studies have attempted to identify the factors that predispose individuals to develop HAV. Biomechanical, genetic and environmental factors have been proposed as potential contributors [6,7,8,9,10,11,12,13,14]; however, the findings have often been contradictory and sometimes inconsistent. Given the wide range of factors potentially involved in the etiology of HAV and the lack of consensus in the literature, determining which mechanisms play a key role in its development remains challenging.

A clearer understanding of the factors that contribute to the onset of HAV is essential to optimize therapeutic strategies, improve risk identification, and facilitate early intervention. In this context, the present systematic review aims to synthesize the available evidence on the etiological factors associated with HAV, thereby providing an updated scientific foundation that may serve as a reference for both clinical research and professional practice. Recent studies continue to support the multifactorial nature of HAV, highlighting the interaction between genetic, morphological, biomechanical, and environmental factors [11,12,14]. Therefore, the primary objective of this systematic review is to examine the etiological factors related to the development of HAV.

## 2. Materials and Methods

### 2.1. Protocol and Registration

This review was conducted in accordance with the PRISMA 2020 (Preferred Reporting Items for Systematic Reviews and Meta-Analyses) guidelines. The protocol was prospectively registered in the International Prospective Register of Systematic Reviews (PROSPERO; registration number: CRD420251004614) and can be accessed at the following link: https://www.crd.york.ac.uk/PROSPERO/myprospero (accessed on 7 March 2025).

### 2.2. Research Question

The research question guiding this review was as follows: in individuals of any age and sex, which etiological factors are associated with the development of HAV?

1. Population (P): Individuals of any age and sex diagnosed with HAV.
2. Exposure (E): Etiological factors.
3. Outcome (O): Development of HAV.
4. Study design: Observational studies, including cross-sectional, case–control, and cohort studies (both retrospective and prospective).

### 2.3. Search Strategy

The search strategy was conducted in the following databases, PubMed (MEDLINE), Embase, Scopus, and Web of Science, from their inception to the most recent update (12 October 2025). The search strategy used in all databases is presented in Table 1. No restrictions were applied regarding language or publication date. The screening process was performed in three stages by two independent reviewers (MMF and PTV), and disagreements were resolved through consensus or consultation with a third reviewer (PMM): (1) removal of duplicates; (2) title and abstract screening using predefined eligibility criteria; (3) full-text assessment of potentially relevant studies to determine final inclusion.

### 2.4. Selection Criteria

The inclusion criteria were as follows: (1) studies involving individuals diagnosed with HAV; (2) articles reporting data on the onset, prevalence or predisposing factors of HAV; (3) studies examining risk factors associated with the development of HAV (biomechanical, genetic, footwear-related or physical activity factors); (4) observational study designs (cohort, case–control and cross-sectional studies). The exclusion criteria were: (1) studies that included other forefoot deformities different from HAV; (2) studies focused exclusively on surgical, orthotic or rehabilitative treatment, unless they provided relevant information regarding etiology or predisposing factors; (3) clinical trials, narrative reviews, letters to the editor, editorials, government reports, clinical guidelines, conference proceedings, conference abstracts, consensus statements or documents lacking methodological description; (4) studies with empirical data that were incomplete or insufficient for the analysis of etiological factors.

### 2.5. Methodological Quality Assessment and Risk of Bias

Methodological quality and risk of bias were assessed using the JBI critical appraisal checklists, adapted to each study design and following the guidelines of the JBI Manual for Evidence Synthesis [15]. The following tools were applied: the JBI Checklist for Analytical Cross-Sectional Studies for cross-sectional designs and the JBI Critical Appraisal Checklist for Case–Control Studies for case–control designs [16]. Although some of the included studies employed descriptive cross-sectional designs, they were evaluated using the analytical cross-sectional checklist, as the JBI Manual does not provide a specific tool for descriptive studies within reviews addressing etiology and risk. This decision was made to ensure methodological consistency and facilitate comparability across scores. Items related to confounder control or statistical analysis were considered not applicable for purely descriptive studies.

Each item was scored as 1 (yes), 0.5 (unclear), or 0 (no). Total scores, quality levels (low, moderate or high), and the percentage (%) of bias were calculated (Table 2 and Table 3). Studies were classified as having low risk of bias (≥75% of total points), moderate risk (50–74%) or high risk (<50%). This approach allows simultaneous assessment of methodological rigor and key sources of bias, including sample representativeness, validity of exposure and outcome measurements, identification of confounding factors, and adequacy of statistical analysis.

The level of evidence for each study was determined according to the OCEBM, 2011 classification, which categorizes research designs into five levels (1–5) based on their ability to establish causal relationships in etiology and risk research [50].

Two reviewers independently performed the quality assessment in duplicate, resolving discrepancies through consensus or consultation with a third reviewer.

## 3. Results

### 3.1. Study Selection

**Figure 1:**
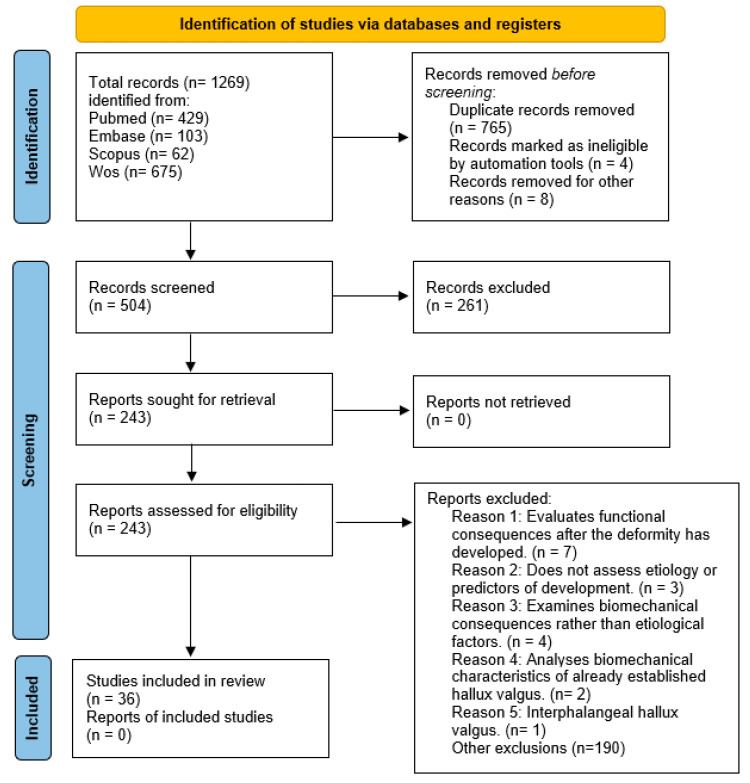
PRISMA flow diagram of study selection.

The initial search identified a total of 1269 articles. After applying the predefined inclusion and exclusion criteria and evaluating the study data, 36 studies were finally selected for analysis (Figure 1).

### 3.2. Study Characteristics

The characteristics of the included studies are presented in Table 4. A total of 20 cross-sectional studies [12,17,18,19,20,21,22,23,24,25,26,27,28,29,30,31,32,33,34,51] and 16 case–control studies [9,14,36,37,38,39,40,41,42,43,44,45,46,47,48,49] were included. The overall sample comprised 14,500 participants, of whom 9085 (62.7%) were women and 5415 (37.3%) were men. The mean age of the total population was 47.3 ± 15.2 years, ranging from pediatric to older adult populations. Most studies reported a predominance of female participants [12,14,20,21,23,24,30,38,39,43,44,46,51]. Four studies were conducted exclusively in women [20,31,41,42], while one study involved only young male participants [37]. The studies were grouped thematically and are discussed in the following sub-sections based on key contributing factors associated with the development of HAV.

#### 3.2.1. Genetic Factors and Family History

Nine studies examined the genetic contribution and the presence of family history in the development of HAV [14,23,31,34,35,41,46,49,51]. Two of these employed observational family-based cross-sectional designs [23,34], whereas seven used case–control designs involving genetic or clinical assessment [14,31,35,41,46,49,51].

The study by Hardy and Clapham [49] was the first to document family history as part of the clinical evaluation, reporting a high proportion of relatives with HAV among affected individuals, although no genetic analyses were performed. This early clinical observation was later confirmed by more systematic investigations. Piqué-Vidal et al. [34] and Zhou et al. [23] assessed familial aggregation of HAV; the former used structured questionnaires with clinical and radiographic confirmation, while the latter applied whole-exome sequencing to identify copy number variations (CNVs). Both studies reported a high prevalence of HAV in first-degree relatives, with Zhou et al. [23] identifying structural genetic alterations specific to affected individuals.

Three studies [31,46,51] recorded family history through standardized clinical interviews, confirming a higher frequency of familial involvement among participants with HAV. In Nery et al. [46], this association was more pronounced in men with greater clinical severity, whereas Okuda et al. [31] found a significant association with affected mothers and/or grandmothers in young women.

Tao et al. [41] investigated genetic susceptibility by genotyping polymorphisms of the vitamin D receptor gene (VDR), identifying a higher prevalence of the TaqI-C and BsmI-A alleles in the HAV group. In the study by Jia et al. [14], whole-exome sequencing was used to detect genetic variants associated with HAV. The authors analyzed more than 18,000 genes and identified pathogenic mutations in COL6A5, COL1A1, HLA-DQB1 and ADAMTSL3. More recently, Kuo et al. [35] conducted a targeted genetic analysis using PCR, demonstrating that the TT genotype of the MTHFR C677T polymorphism is significantly associated with an increased risk of developing HAV, whereas A1298C showed no association.

Two studies [46,51] performed sex-stratified analyses to explore sex-related differences in familial aggregation, and one study focused on a juvenile population, allowing for the examination of hereditary influence at early developmental stages [31].

#### 3.2.2. First-Ray Bone Morphology and Multiplanar Deformity

Nine studies examined the bone morphology of the first ray and the multiplanar component of HAV using imaging techniques in adult populations [24,27,38,39,42,43,47,48,49].

Of these, seven were case–control studies [38,39,42,43,47,48,49] primarily assessing morphometric parameters through weightbearing radiographs [43,47,48,49] or computed tomography (CT/WBCT) [38,39,42]. The variables analyzed included the intermetatarsal angle (IMA), relative first metatarsal length, geometric asymmetry of the proximal phalanx (medial–lateral diameter difference), as well as frontal-plane rotation of the first ray and the hallux. All studies reported significant between-group differences, with higher angular deviation, increased osseous pronation, or greater structural asymmetry in individuals with HAV.

Two studies employed a cross-sectional design [24,27]. Bu et al. [24] analyzed the morphology of the medial cuneiform using digital radiography and identified angular variations in the Metatarsocuneiform Angle (MCA), Metatarsus Adductus Angle (MAA), and Proximal Metatarsal Articular Angle (PMAA) associated with HAV. Manceron et al. [27] used dynamic ultrasound to quantify the multiplanar mobility of the first ray and the first tarsometatarsal joint (1st TMTJ) exclusively in HAV feet, observing greater displacement in cases with more advanced deformity.

#### 3.2.3. Intrinsic Biomechanical Factors

Eight studies examined intrinsic biomechanical factors associated with the development of HAV, grouped into first-ray hypermobility, flatfoot/pronation, and intrinsic foot muscle function [9,17,18,27,37,40,42,51]. Four of these were case–control studies [9,37,40,42], three were analytical cross-sectional studies [17,18,51], and one was a descriptive cross-sectional study [27].

With regard to first-ray hypermobility, two studies evaluated its multiplanar motion [27,42]. Kimura et al. [42] compared individuals with HAV and controls using WBCT, identifying significantly greater first-ray mobility in dorsiflexion, inversion and adduction in the HAV group. Conversely, Manceron et al. [27] analyzed only HAV-affected feet using dynamic ultrasound, observing increased motion at the 1st TMTJ in cases presenting with more advanced deformities.

Four studies investigated parameters related to rearfoot alignment and arch structure [17,18,37,51]. Atbaşı et al. [37] analyzed weightbearing radiographs in young adult males and observed a reduced calcaneal pitch and increased lateral talocalcaneal angle in the HAV group. Choi et al. [18] employed an artificial intelligence-based classification system to assess flatfoot severity using radiographs and found a significant association between flatfoot and increased HAV severity. Nguyen et al. [51], in an older general population, used MatScan baropodometry and reported that flatfoot was associated with a higher likelihood of HAV in men, although this association was not observed in women.

Two studies investigated the activity of the abductor hallucis (AbdH) muscle using surface electromyography [9,40]. Arinci Incel et al. [9] observed significantly lower AbdH activity in individuals with HAV during voluntary hallux abduction tasks. Similarly, Mortka et al. [40] reported reduced motor unit action potential amplitude in the HAV group, although this reduction did not correlate with radiographic severity.

#### 3.2.4. Anthropometric and Demographic Factors

Six studies evaluated the influence of anthropometric and demographic characteristics on the development of HAV [12,20,21,30,46,51]. Five of these were analytical cross-sectional studies [12,20,21,30,51], and one employed a case–control design [46].

Three studies [21,30,51] included sex- and age-stratified analyses. Nguyen et al. [51] reported a higher prevalence of HAV in women than in men and found sex-specific associations when analyzing BMI and flatfoot. Nishimura et al. [30] also identified a higher frequency of HAV in older women, with a mean age of 75.5 ± 6.4 years. Nakao et al. [21], using multivariable analysis with feature selection (SVM-RFE), identified age, female sex and body weight as key variables distinguishing participants with HAV from those without.

Two studies analyzed foot morphological variables and their association with HAV [12,20]. Martín-Casado et al. [12] assessed foot length and width, heel width and arch height using a 3D scanner, reporting that greater foot length and wider heel width were associated with HAV. Liu et al. [20] examined adolescent athletes and found higher prevalence and severity in females, as well as an increased risk associated with greater weekly training load.

Finally, in the study by Nery et al. [46], which included adult participants of both sexes, a more deviated first ray and greater HAV severity were more frequent in men, despite the overall lower prevalence in this group.

#### 3.2.5. Lower-Limb Alignment and Biomechanics

Two analytical cross-sectional studies evaluated the influence of lower-limb characteristics on the development of HAV [36,45]. In the study by O’Reilly et al. [36], ankle dorsiflexion and gastrocnemius contracture were measured using the Silfverskiöld test, revealing reduced dorsiflexion and a higher prevalence of gastrocnemius tightness in individuals with HAV compared with controls. Steinberg et al. [45] assessed proximal alignment using goniometric measurements (Q-angle, tibiofemoral angle, rearfoot angle) and recorded generalized hypermobility through the Beighton Score, a validated nine-item scale used to quantify joint laxity. They found significantly higher values among participants with HAV. Both studies demonstrated proximal biomechanical differences between individuals with and without HAV.

#### 3.2.6. Extrinsic, Metabolic and Lifestyle Factors

Six analytical cross-sectional studies evaluated the relationship between footwear characteristics and hallux valgus angles across different developmental stages [25,26,28,29,32,44].

In pediatric populations, Klein et al. [32] used 3D scanning combined with a device specifically designed to measure internal shoe length, reporting higher hallux valgus angle (HVA) values in children whose footwear provided insufficient toe allowance. González-Elena et al. [28] combined direct anthropometric foot and shoe measurements with weightbearing podoscopy, identifying significant correlations between reduced shoe allowance and higher HVA within age- and sex-specific subgroups. Puszczalowska-Lizis et al. [29] used podoscopy and digital measurements of shoe “functional excess,” finding significantly greater HVA values in children wearing shoes that were too short. Similarly, Kinz et al. [26] digitally evaluated internal shoe length and HVA and observed a high prevalence of short footwear and higher HVA values in such cases, while habitual barefoot walking was associated with smaller angles.

In an adult population, Menz et al. [44] used a validated retrospective questionnaire featuring line drawings of forefoot shapes. Their findings indicated a significantly higher likelihood of HAV among participants who had worn narrow toe boxes during their 20s and 30s. Additionally, Dittmar et al. [25], through physical anthropology of skeletal remains, found a greater prevalence of HAV during historical periods associated with tight, pointed footwear compared to barefoot populations.

Finally, Liu et al. [19] investigated the relationship between sedentary behavior and HAV risk using a genetics-based statistical approach known as Mendelian Randomization, drawing data from large European population cohorts. Sedentary behavior was quantified using leisure screen time. The authors found that higher screen time was significantly associated with an increased risk of developing HAV.

### 3.3. Risk of Bias in the Included Studies

The methodological quality of the 36 included studies was generally adequate. According to the scores obtained using the JBI tools, 31 studies (87.1%) demonstrated a low risk of bias, whereas five studies (12.9%) showed a moderate risk, and none were classified as having a high risk of bias.

By study design, cross-sectional studies showed a predominantly low risk of bias (17/18; 94.4%), with only one study rated as moderate. In contrast, case–control studies presented 10 out of 13 (76.9%) with low risk and three out of 13 (23.1%) with moderate risk.

The items with the highest risk of bias were related to the lack of control for confounding factors, insufficient reporting on the reliability and validity of measurements, and the use of non-probabilistic sampling methods. In contrast, the best-performing items were the clarity of inclusion criteria, the adequate measurement of exposures and outcomes, and the use of appropriate statistical analyses in most analytical studies. The detailed scoring for each study is presented in Table 2.

## 4. Discussion

The primary objective of this systematic review was to examine the etiological factors associated with the development of HAV. The findings are associated with the current understanding of HAV as a complex and multifactorial deformity influenced by the interaction of several intrinsic and extrinsic factors. To our knowledge, this work represents the first systematic review to comprehensively synthesize evidence across all these domains, integrating genetic, morphological, biomechanical, anthropometric, and extrinsic factors within a single etiological framework. This integrative perspective provides a clinically oriented synthesis of the etiological factors involved in HAV and may support more individualized preventive and therapeutic strategies.

### 4.1. Genetic Factors and Family History

The results suggest that genetic inheritance may represent one of the principal etiological factors associated with HAV. Classic studies by Hardy and Clapham [49] and Piqué-Vidal et al. [34] already demonstrated strong familial aggregation, with a prevalence of up to 90% in first-degree relatives. Subsequent research [31,46,51] corroborated this association, showing that a positive family history significantly increases the likelihood of developing HAV.

Recent molecular evidence expands this knowledge. Jia et al. [14], through whole-exome sequencing, identified variants in genes related to collagen synthesis and the extracellular matrix (COL6A5, COL1A1), immune modulation (HLA-DQB1) and connective tissue organization (ADAMTSL3), suggesting a genetic susceptibility that affects the capsuloligamentous stability of the first ray. These findings indicate that HAV may be understood, at least in part, as a disorder linked to connective tissue integrity and extracellular matrix signaling pathways, rather than solely as an acquired forefoot deformity.

Tao et al. [41] reported higher frequencies of the TaqI-C and BsmI-A alleles of the VDR in individuals with HAV, reinforcing the hypothesis of a multifactorial genetic susceptibility modulated by hormonal and metabolic factors. This line of evidence was further expanded by Zhou et al. [23], who identified CNVs in genes related to the cytochrome P450 pathway (CYP2D6, CYP2D7) as well as in immunoregulatory genes (HLA-H, HCG4B), suggesting that HAV may have an immunometabolic basis affecting cartilage homeostasis and local inflammatory responses.

The most recent study, conducted by Kuo et al. [35], provides additional evidence by identifying that individuals carrying the TT genotype of the MTHFR C677T polymorphism have a significantly increased risk of developing HAV. This finding suggests a potential link between folate metabolism, DNA methylation and individual susceptibility to the deformity, introducing a biologically plausible pathway that aligns with the previously described genetic complexity.

Consistent with the clinical observations by Nery et al. [46] and Okuda et al. [31], these genetic alterations appear to manifest differently between men and women and may exert their influence from early ages. Overall, the current genetic evidence supports the view that HAV has a complex hereditary basis, with contributions from both connective tissue structure and immunometabolic mechanisms.

### 4.2. Bone Morphology and the Multiplanar Component

The literature consistently describes HAV as a multiplanar deformity of the first ray. Imaging studies consistently indicate that structural and rotational changes in the first ray are associated with HAV [24,27,38,39,42,43,48].

Cruz et al. [38], using WBCT, quantified an average first-ray pronation of 15.3° in the HAV group versus 3.4° in controls, and observed significant medial rotation of the hallux, confirming the three-dimensional nature of the deformity. Kimura et al. [42] reported a similar pattern, additionally describing dorsal and medial translation of the first metatarsal. Bu et al. [24] highlighted the involvement of the medial cuneiform, identifying angular variations (MCA, PMAA, MAA) that predispose to medial displacement of the first metatarsal.

Similarly, Mancuso et al. [48] and Munuera et al. [47] provided evidence regarding the role of longitudinal bone morphology, showing that individuals with HAV present a longer first metatarsal than controls. Mancuso et al. [48] further reported that this increased length is often accompanied by a more rounded metatarsal head. Later, Munuera et al. [47] confirmed that, in early stages, individuals with HAV also exhibit a longer hallux. These features, identifiable in the early stages of the deformity, may constitute key predisposing traits that precede the multiplanar deviation of the first ray, reinforcing the notion that certain anatomical configurations may predispose to the deformity independently of functional biomechanical mechanisms.

Manceron et al. [27], through dynamic ultrasound, observed greater multiplanar displacement of the 1st TMTJ in more severe cases, supporting the view that first-ray instability is not only structural but also functional.

Collectively, these findings suggest that the bone morphology of the first metatarsal and medial cuneiform may serve as a primary anatomical predisposition for the development of HAV, with secondary biomechanical factors influencing the severity and progression of the deformity.

### 4.3. Intrinsic Biomechanical Factors

Intrinsic biomechanical alterations appear to play a relevant role in the development and progression of HAV, particularly those affecting first-ray stability, foot pronation, and intrinsic muscle function.

First-ray hypermobility was identified as a key element [27,42]. Kimura et al. [42] reported significantly greater dorsal and medial displacement of the first metatarsal in individuals with HAV, while Manceron et al. [27] confirmed through dynamic ultrasound that mobility of the 1st TMTJ progressively increased with greater deformity severity. Chen et al. [17] further validated this functional impairment using dynamic biomechanical assessment and baropodometry, demonstrating a redistribution of plantar load toward the first metatarsal head and an increase in medial pressure mechanisms that perpetuate the progressive displacement of the hallux and the first ray.

Regarding rearfoot alignment and arch structure, Atbaşı et al. [37] found reduced calcaneal pitch and increased lateral talocalcaneal angle values in young men with HAV, indicative of flatfoot. Choi et al. [18] used artificial intelligence algorithms applied to radiographs to quantify flatfoot severity, identifying a significant association with the presence of HAV. In older adults, Nguyen et al. [51] demonstrated that flatfoot was associated with a higher likelihood of HAV in men but not in women, suggesting sex-specific biomechanical patterns.

Additionally, electromyographic studies by Arinci Incel et al. [9] and Mortka et al. [40] revealed significantly reduced activity of the abductor hallucis muscle, an important stabilizer of the medial column reinforcing the role of intrinsic musculature in the progression of the deformity.

Together, these findings support the notion that HAV is associated with medial instability, excessive pronatory loading and neuromuscular deficits, which contribute to the progressive displacement of the hallux and the first ray.

### 4.4. Anthropometric and Demographic Factors

Female sex and older age are the primary demographic factors associated with HAV [21,22,30,51]. Jung et al. [22] found that being female, over the age of 50, and having a low medial longitudinal arch significantly increase the likelihood of developing HAV, highlighting the interplay between hormonal, structural, and mechanical influences.

Liu et al. [20] examined adolescent athletes and found a higher prevalence and severity of HAV in females, as well as a positive relationship between weekly training load and the deformity. This suggests that repetitive stress on a vulnerable forefoot may precipitate HAV at an early age.

From an anthropometric perspective, Martín-Casado et al. [12] identified associations between greater foot length, wider heel width, and reduced arch height with the presence of HAV. Additionally, Munuera et al. [47] reported that individuals with early-stage HAV exhibited a proportionally longer first metatarsal and hallux, suggesting that particular longitudinal forefoot configurations may form part of a presuppositional anthropometric profile.

Collectively, these findings suggest an influence of morphological phenotype and load distribution patterns on susceptibility to HAV.

### 4.5. Lower-Limb Alignment and Biomechanics

The influence of lower-limb alignment and global postural patterns also appear to contribute significantly to the onset and progression of HAV. The limitations in ankle dorsiflexion and gastrocnemius tightness described by O’Reilly et al. [36] may promote a distal compensatory pattern characterized by increased subtalar pronation and higher medial loading during stance phase conditions that may amplify the deviating forces acting on the first ray.

Similarly, the proximal alignment alterations and generalized hypermobility observed by Steinberg et al. [45], including an increased Q-angle and greater ligamentous laxity, may compromise the stability of the tarsometatarsal complex, facilitating progressive medial deviation of the first metatarsal.

These findings indicate that specific patterns of proximal alignment and mobility may act as biomechanical modulators that favor or accelerate the manifestation of HAV in predisposed individuals.

### 4.6. Extrinsic, Metabolic, and Lifestyle Factors

The use of narrow or ill-fitting footwear remains one of the most frequently cited extrinsic factors associated with HAV, particularly during childhood and early adulthood. In children, Klein et al. [32], Puszczalowska-Lizis et al. [29], González-Elena et al. [28] (2021), and Kinz et al. [26] demonstrated a significant correlation between wearing small or narrow shoes and higher HVA values, suggesting that forefoot compression during growth influences hallux alignment.

In adults, Menz et al. [44] found that wearing narrow toe-box footwear between the ages of 20 and 39 doubled the risk of developing HAV (OR = 2.7; 95% CI: 1.46–5.00). From a historical perspective, Dittmar et al. [25] demonstrated through osteological analyses that the prevalence of HAV was higher in populations that wore tight or pointed footwear compared with those that went barefoot.

Collectively, these findings support the notion that footwear acts as a modulating factor capable of precipitating or exacerbating an underlying anatomical predisposition rather than serving as a primary cause of HAV.

More recently, Liu et al. [19] established a causal relationship between sedentary behavior and HAV development, partially mediated by low serum calcium levels. This suggests that reduced physical activity and metabolic imbalances may affect muscle function and bone integrity, indirectly contributing to the onset or progression of HAV. Furthermore, the authors propose that lifestyle modifications and the optimization of mineral metabolism should be considered in future preventive and rehabilitative strategies.

Genetic variations may produce a more unstable articular architecture or an altered inflammatory response; on this substrate, excessive pronation, hypermobility, and restrictive footwear act as mechanical triggers. This model explains the gradual progression of HAV from functional instability to structural deformity consistent with the theory of progressive medial instability described by Coughlin et al. [33] and Okuda et al. [31].

**Figure 2:**
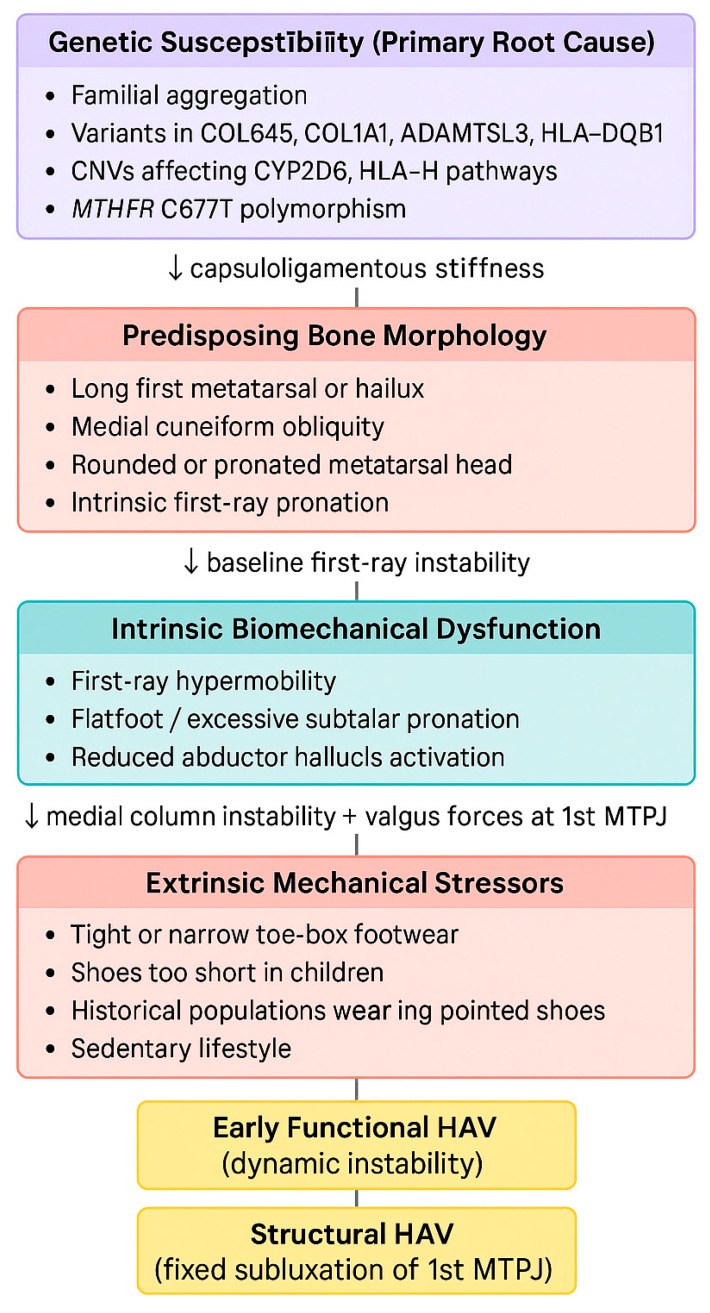
Conceptual model of the multifactorial pathogenesis and progression of hallux abducto valgus (HAV). Genetic susceptibility may predispose to altered articular morphology, reduced capsuloligamentous stiffness, and intrinsic biomechanical dysfunction of the first ray. In this vulnerable substrate, excessive pronation, hypermobility, and extrinsic mechanical stressors, particularly restrictive footwear, contribute to medial column instability and valgus loading at the first metatarsophalangeal joint (1st MTPJ), promoting progression from early functional HAV to structural deformity.

A conceptual model of this etiologic pathway is presented in Figure 2.

From a clinical perspective, understanding the multifactorial etiological model of HAV may support a more precise and personalized therapeutic approach. The identification of risk profiles based on the interaction between genetic predisposition, bone morphology, biomechanical patterns, and extrinsic factors opens the door to early preventive strategies and to treatment selection directed at the predominant mechanisms in each patient. This integrative perspective may contribute to a more individualized form of medicine, aimed not only at correcting the deformity, but also at modifying the factors that condition its onset and progression.

### 4.7. Limitations

This review presents several limitations that must be considered when interpreting the findings. Although most studies demonstrated a low risk of bias according to the JBI tool, the overall evidence is constrained by methodological heterogeneity, the predominantly cross-sectional design of many studies, and the limited inclusion of confounding variables. Most included studies were cross-sectional or case–control, which allow the identification of associations but do not establish causality or determine the temporal sequence between exposure and HAV onset. The lack of longitudinal or cohort studies therefore represents the main limitation of this review. Additionally, some studies used small samples or highly specific populations, which may restrict the generalizability of the findings.

In addition, although most studies showed a low risk of bias according to the JBI assessment, the overall level of evidence remains limited due to the predominance of observational designs. Consequently, the current evidence does not allow for the establishment of strong clinical recommendations or the development of robust clinical guidelines, highlighting the need for well-designed longitudinal and prospective studies.

## 5. Conclusions

HAV is a multifactorial deformity associated with the interaction between genetic susceptibility, predisposing bony morphology, biomechanical dysfunction, and environmental factors. Current evidence supports a dynamic and three-dimensional etiological model in which intrinsic factors determine structural vulnerability, while extrinsic factors act as triggers. Understanding this interaction is essential for developing individualized preventive and therapeutic strategies focused on functional correction and early detection in predisposed individuals. This integrative etiological framework provides a conceptual basis for future longitudinal research and improved patient-specific risk assessment.

## Figures and Tables

**Table 1:** Search strategy used in the electronic databases.

Database	Search Terms
All databases	(hallux valgus OR hallux abductovalgus) NOT (hallux limitus OR hallux rigidus OR hallux varus) AND (etiolog OR aetiology OR risk factors OR factor OR caus* OR pathogenesis OR genetics OR congenital OR famil* OR hered* OR inherit* OR hormonal OR relaxin OR shoe* OR shoewear OR footwear OR osteoarthritis OR arthritis OR rheumat* OR hipermobility OR first ray OR first metatarsal head OR peroneus longus OR intrinsic musculature OR extrinsic musculature OR biomechanics) NOT (surgery OR osteotomy OR treatment OR therap*).

**Table 2:** Methodological quality assessment of the included studies using the JBI critical appraisal checklists for cross-sectional and case–control designs.

Author	Study Type	1	2	3	4	5	6	7	8	9	10	11	Total
Chen et al. [17]	Cross-Sectional Studies	1	0.5	1	1	0.5	0	1	1				6/8
Choi et al. [18]		1	1	1	1	0.5	0.5	1	1				7/8
Liu S et al. [19]		1	1	1	1	1	1	1	1				8/8
Liu Z et al. [20]		1	1	1	1	0.5	0.5	1	1				7/8
Nakao et al. [21]		1	1	1	1	0.5	0.5	1	1				7/8
Jung et al. [22]		1	1	1	1	0.5	0.5	1	1				7/8
Martín-Casado et al. [12]		1	1	1	1	0.5	0.5	1	1				7/8
Zhou et al. [23]		1	0.5	1	1	0.5	0	1	0.5				5.5/8
Bu et al. [24]		1	1	1	1	0.5	0.5	1	1				7/8
Dittmar et al. [25]		1	1	1	1	1	1	1	1				8/8
Kinz et al. [26]		1	1	1	1	0.5	1	1	1				7/8
Manceron et al. [27]		1	1	1	1	0.5	0.5	1	1				7/8
González-Elena et al. [28]		1	1	1	1	0.5	0	1	1				6.5/8
Puszczalowska-Lizis et al. [29]		1	1	1	1	0.5	0	1	1				6.5/8
Nishimura et al. [30]		1	1	1	1	1	1	0.5	1				7.5/8
Okuda et al. [31]		1	1	1	1	1	1	1	1				8/8
Klein et al. [32]		1	1	1	1	0.5	0	1	1				6.5/8
Coughlin et al. [33]		1	1	1	1	0.5	0.5	1	1				7/8
Piqué-Vidal et al. [34]		1	1	1	1	0.5	1	1	1				7.5/8
Kuo et al. [35]		1	1	1	1	1	1	1	1	0.5	1		9.5/10
Jia et al. [14]		1	1	1	0.5	0.5	0.5	1	1	0	1		7.5/10
O’Reilly et al. [36]	Case–Control Studies	0.5	1	1	1	1	1	0.5	0.5	0	1		6.5/10
Atbasi et al. [37]		0.5	1	1	1	1	0.5	0	1	0.5	1		7.5/10
Cruz et al. [38]		1	1	1	1	1	1	0.5	0.5	0	1		8/10
Gómez Galván et al. [39]		1	1	1	1	1	1	1	0.5	0	1		8.5/10
Mortka et al. [40]		1	1	1	1	1	1	1	0.5	0	1		8.5/10
Tao et al. [41]		1	1	1	1	1	1	0.5	0.5	0	1		8/10
Kimura et al. [42]		1	1	1	1	1	1	1	1	0	1		9/10
Perez Boal et al. [43]		1	1	1	1	1	1	1	1	0	1		9/10
Menz et al. [44]		1	0.5	1	0.5	1	0	0	1	1	1		7/10
Steinberg et al. [45]		1	1	1	1	1	1	0.5	0.5	0	1		8/10
Nery et al. [46]		1	1	1	0.5	0	1	0	1	1	1		7.5/10
Munuera et al. [47]		1	1	1	1	1	0.5	0.5	1	0	1		8/10
Arinci Incel et al. [9]		1	1	1	1	1	1	0.5	0.5	0	1		8/10
Mancuso et al. [48]		0	0	1	1	1	0	0	1	0.5	1		5.5/10
Hardy et al. [49]		0.5	1	1	0.5	1	1	0.5	0	0	1		6.5/10

1: Yes, 0: No, 0.5: Unclear, gray: Not applicable. Case–control studies: 8–10 = high quality; 5–7 = moderate quality; ≤4 = low quality. Cross-sectional studies: 6–8 = high quality; 4–5 = moderate quality; ≤3 = low quality.

**Table 3:** Risk of bias classification of the included studies based on the JBI critical appraisal scores.

Study	Checklist Tools	JBI Critical Appraisal	
Chen et al. [17]	JBI Checklist for Analytical Cross-Sectional Studies	6/8 (75%)	Low
Choi et al. [18]	JBI Checklist for Analytical Cross-Sectional Studies	7/8 (87.5%)	Low
Liu S et al. [19]	JBI Checklist for Analytical Cross-Sectional Studies	8/8 (100%)	Low
Liu Z et al. [20]	JBI Checklist for Analytical Cross-Sectional Studies	7/8 (87.5%)	Low
Nakao et al. [21]	JBI Checklist for Analytical Cross-Sectional Studies	7/8 (87.5%)	Low
Jung et al. [22]	JBI Checklist for Analytical Cross-Sectional Studies	7/8 (87.5%)	Low
Martín-Casado et al. [12]	JBI Checklist for Analytical Cross-Sectional Studies	7/8 (87.5%)	Low
Zhou et al. [23]	JBI Checklist for Analytical Cross-Sectional Studies	5.5/8 (68.8%)	Moderate
Bu et al. [24]	JBI Checklist for Analytical Cross-Sectional Studies	7/8 (87.5%)	Low
Dittmar et al. [25]	JBI Checklist for Analytical Cross-Sectional Studies	8/8 (100%)	Low
Kinz et al. [26]	JBI Checklist for Analytical Cross-Sectional Studies	7/8 (87.5%)	Low
Manceron et al. [27]	JBI Checklist for Analytical Cross-Sectional Studies	7/8 (87.5%)	Low
González-Elena et al. [28]	JBI Checklist for Analytical Cross-Sectional Studies	6.5/8 (81.3%)	Low
Puszczalowska-Lizis et al. [29]	JBI Checklist for Analytical Cross-Sectional Studies	6.5/8 (81.3%)	Low
Nishimura et al. [30]	JBI Checklist for Analytical Cross-Sectional Studies	7.5/8 (87.5%)	Low
Okuda et al. [31]	JBI Checklist for Analytical Cross-Sectional Studies	8/8 (100%)	Low
Klein et al. [32]	JBI Checklist for Analytical Cross-Sectional Studies	6.5/8 (81.3%)	Low
Coughlin et al. [33]	JBI Checklist for Analytical Cross-Sectional Studies	7/8 (87.5%)	Low
Piqué-Vidal et al. [34]	JBI Checklist for Analytical Cross-Sectional Studies	7.5/8 (87.5%)	Low
Kuo et al. [35]	JBI Critical Appraisal Checklist for Case–Control Studies	9.5/10 (95%)	Low
Jia et al. [14]	JBI Critical Appraisal Checklist for Case–Control Studies	7.5/10 (75%)	Low
O’Reilly et al. [36]	JBI Critical Appraisal Checklist for Case–Control Studies	6.5/10 (65%)	Moderate
Atbasi et al. [37]	JBI Critical Appraisal Checklist for Case–Control Studies	7.5/10 (75%)	Low
Cruz et al. [38]	JBI Critical Appraisal Checklist for Case–Control Studies	8/10 (80%)	Low
Gómez Galván et al. [39]	JBI Critical Appraisal Checklist for Case–Control Studies	8.5/10 (85%)	Low
Mortka et al. [40]	JBI Critical Appraisal Checklist for Case–Control Studies	8.5/10 (85%)	Low
Tao et al. [41]	JBI Critical Appraisal Checklist for Case–Control Studies	8/10 (80%)	Low
Kimura et al. [42]	JBI Critical Appraisal Checklist for Case–Control Studies	9/10 (90%)	Low
Perez Boal et al. [43]	JBI Critical Appraisal Checklist for Case–Control Studies	9/10 (90%)	Low
Menz et al. [44]	JBI Critical Appraisal Checklist for Case–Control Studies	7/10 (70%)	Moderate
Steinberg et al. [45]	JBI Critical Appraisal Checklist for Case–Control Studies	8/10 (80%)	Low
Nery et al. [46]	JBI Critical Appraisal Checklist for Case–Control Studies	7.5/10 (75%)	Low
Munuera et al. [47]	JBI Critical Appraisal Checklist for Case–Control Studies	8/10 ((80%)	Low
Arinci Incel et al. [9]	JBI Critical Appraisal Checklist for Case–Control Studies	8/10 (80%)	Low
Mancuso et al. [48]	JBI Critical Appraisal Checklist for Case–Control Studies	5.5/10 (55%)	Moderate
Hardy et al. [49]	JBI Critical Appraisal Checklist for Case–Control Studies	6.5/10 (65%)	Moderate

**Table 4:** Main characteristics of the included studies, including study design, level of evidence, sample characteristics, etiological factors evaluated, methods, main results, and conclusions.

Author (Year), Study Design and Country	Level of Evidence (OCEBM)	Objective	Sample Size (N)	Participants	Risk/Etiologic Factors	Methods	Results	Conclusions
Chen et al. [17] (2025). Analytical cross-sectional study. China. Taiwán.	4	To analyze the correlation between HAV severity and the presence of metatarsus adductus.	198 adults	F = 108 M = 90 49.6 ± 10.4 years.	HAV severity and metatarsus adductus.	Weightbearing AP radiographs (HVA, IMA, MA); PACS software; Manchester classification.	Significant correlation between HAV severity and metatarsus adductus (r = 0.42; *p* < 0.001).	Metatarsus adductus may act as an anatomical predisposing factor for increased HAV severity.
Choi et al. [18] (2025). Analytical cross-sectional study. South Korea.	3b	To analyze the correlation between HAV and flatfoot using artificial intelligence.	520 adults	F = 308 M = 212 51.7 ± 12.3 years.	Relationship between HVA, IMA, and calcaneal–flatfoot angles.	Convolutional neural network-assisted radiographs with automatic angle measurement.	Significant positive correlation between HAV severity and the flatfoot angle.	The coexistence of HAV and flatfoot suggests a shared biomechanical pattern.
Liu S. et al. [19] (2025). Analytical cross-sectional study. China.	2b	To evaluate the causal relationship among sedentary behavior, calcium homeostasis, and HAV risk.	Aggregated GWAS data from multiple European cohorts	NA.	Sedentary behavior, serum calcium levels, bone metabolism.	Two-sample Mendelian randomization using GWAS data; two-step mediation models.	Sedentary behavior increases HAV risk (OR = 1.19); serum calcium reduces risk (OR = 0.75).	Sedentary behavior is a risk factor partially mediated by calcium metabolism.
Kuo et al. [35] (2025). Case–control study. Taiwán.	3b	To investigate the association between MTHFR gene polymorphisms (rs1801133 and rs1801131) and the risk of developing HAV in a Taiwanese population.	750 adults	F = 600 M = 150 50.7 ± 15.3 years (150 with HAV, 600 controls).	Genetic polymorphisms: MTHFR C677T (rs1801133) and MTHFR A1298C (rs1801131).	HAV diagnosis using a standardized foot diagram (>15° hallux deviation). DNA extraction from leukocytes; genotyping via PCR-RFLP; statistical analysis with adjusted ORs, chi-square tests and genetic trend analysis.	The TT genotype of MTHFR rs1801133 was associated with a 2.57-fold increased risk of HAV. The CT genotype did not increase risk. The rs1801131 polymorphism showed no association with HAV. The association did not vary by sex, age, weight, height or BMI.	The TT genotype of MTHFR rs1801133 is a genetic risk marker for HAV.
Liu Z. et al. [20] (2024). Analytical cross-sectional study. Japan.	3b	To determine the prevalence of HAV and associated factors in adolescent dancers.	275 adolescent athletes	F = 202 M = 73 14.5 ± 2.1 years.	Sex, age, weekly training time and years of practice.	Questionnaires, plantar photographs, logistic regression analysis.	Higher prevalence and severity of HAV were observed in females and in those with greater weekly training loads.	Female sex and prolonged training are key risk factors for HAV in adolescents.
Jung et al. [22] (2023). Analytical cross-sectional study. South Korea.	3b	To identify the factors that contribute to HAV using a decision tree model.	864 adults	F = 586 M = 278 54.2 ± 13.6 years.	Age, sex, BMI, arch height.	Weightbearing dorsoplantar radiographs; 3D foot scanner for arch height; demographic questionnaires.	Female sex and older age were the most influential predictors. BMI and arch height also contributed to the model.	The combination of advanced age, female sex and higher arch height increases the likelihood of HAV.
Martín-Casado et al. [12] (2023). Analytical cross-sectional study. Ecuador.	3b	To evaluate morphological foot differences across BMI categories and identify HAV risk factors in children and adolescents.	1678 children 3356 feet	F = 844 M = 834 10.54 ± 3.60 normal weight, 11.21 ± 3.64 overweight, 9.34 ± 3.20 obesity.	Age, sex, foot length, metatarsal width, heel width, arch height and BMI.	INFOOT 3D scanner; standardized marking of 13 anatomical points; HVA calculation; BMI classification.	Overweight and obese children had longer and wider feet. Age, foot length and heel width were risk factors; male sex, metatarsal width and higher arch height were protective.	Excess weight is associated with greater foot length and width. Age, foot length and heel width increase HAV risk, while a higher arch and wider forefoot reduce it.
Nakao et al. [21] (2023). Analytical cross-sectional study. Japan.	3b	To identify individual factors associated with HAV and rank their importance using a machine learning model (SVM-RFE).	864 adults 1728 feet	F = 511 M = 353 51.6 ± 21 years.	Age, sex, body weight, instep height, foot length, BMI, arch height, history of foot pain/injury, and exercise habits.	Manchester Scale; Takei Corp measurement device; Support Vector Machine–Recursive Feature Elimination with cross-validation; Student’s t, χ^2^, Pearson’s/Spearman’s correlations.	Age, sex and body weight were the most relevant features. HAV was more common in women and increased significantly in individuals aged 45–64. No significant correlation with body weight or arch height.	HAV is primarily associated with age and sex, being more prevalent in females and in middle-aged adults. Body weight and arch height showed limited influence.
Zhou et al. [23] (2023). Analytical cross-sectional study. China.	4	To assess the relationship between TMT joint rotation and HAV severity using weightbearing CT.	80 adults 122 feet	F = 68 M = 12 49.3 ± 12.6 years.	TMT rotation angle, 1st metatarsal pronation, IMA and HVA.	Weightbearing CT (CurveBeam pedCAT); 3D measurements using Disior Bonelogic software; HAV severity classified by HVA values.	TMT rotation angle increased significantly with HAV severity. Moderate and severe HAV showed greater pronation and larger TMT angle.	TMT joint rotation is strongly correlated with HAV severity and may represent a contributing morphological factor.
Manceron et al. [27] (2022). Analytical cross-sectional study. France.	4	To evaluate the correlation between 1st TMT joint mobility and HAV severity.	38 adults 50 feet	F = 33 M = 5 55.3 ± 12.1 years.	TMT mobility and radiographic deformity severity.	Weightbearing radiographs (HVA, IMA); fluoroscopic assessment of TMT mobility; statistical correlations.	Greater TMT mobility was associated with more severe deformity.	TMT hypermobility is associated with increased HAV severity and may play an etiological role.
Bu et al. [24] (2022). Analytical cross-sectional study. China.	4	To analyze the relationship between distal articular inclination of the medial cuneiform and the presence or severity of HAV on weightbearing radiographs.	534 adults 679 feet	F = 314 M = 2020 36.0 ± 16.0 years.	HVA, IMA, MAA, MCA, DMCA, PMAA, and TMT joint morphology; age and sex.	Weightbearing AP radiographs; measurements using CAD2012 software; statistical analysis with SPSS 19.0 (*t*-test, Wilcoxon, χ^2^, multivariate linear regression).	The HAV group showed significantly higher IMA, MAA and MCA values (*p* < 0.05), with no differences in DMCA or TMT morphology. Regression analysis identified age, MCA and DMCA as predictors of HVA, and age, MAA, MCA and DMCA as predictors of IMA.	Medial cuneiform inclination (particularly MCA) is associated with HAV severity. DMCA remains constant and is not a reliable severity indicator; MCA may serve as a characteristic angle for assessing HAV severity.
Dittmar et al. [25] (2024). Analytical cross-sectional study. Germany.	4	To explore the relationship between forefoot alignment and HAV severity.	502 adults	F = 292 M = 210 52.3 ± 14.2 years.	Load distribution, foot morphology, forefoot angular parameters.	3D foot scanner and baropodometric assessment.	Increased pronation and higher intermetatarsal angles were associated with more severe HAV.	Alterations in forefoot morphology and pronation patterns contribute to HAV development and progression.
Kinz et al. [26] (2021). Analytical cross-sectional study. Austria.	4	To evaluate the relationship between inadequate shoe length and HVA in preschool children, and to analyze the effect of habitual barefoot walking.	620 children	F = 304 M = 316. -	Insufficient shoe length (internal and external), HVA, frequency of barefoot walking.	Interior shoe-length measuring device; foot scanner with digital HVA measurement; shoe classification (adequate/too short/excessively short); regression models.	75.5% wore shoes that were too short externally, and 84,6% wore shoes too short internally. HVA was significantly higher in children wearing excessively short shoes. Barefoot walking was associated with lower HVA values.	Insufficient shoe length is strongly associated with increased HVA in preschool children. Regular barefoot walking may act as a protective factor.
Jia et al. [14] (2021). Case–control study. China.	3b	To identify rare genetic variants and de novo mutations associated with HAV using whole-exome sequencing.	164 adults	F = 113 M = 51 46 ± 12 years (68 HAV, 96 controls).	Genes involved in collagen structure, extracellular matrix integrity and immune regulation.	Bioinformatic analysis of rare variants including functional prediction and gene-enrichment analyses (GO and KEGG).	HAV cases presented rare variants in COL6A5, COL1A1, HLA-DQB1 and ADAMTSL3, all involved in collagen synthesis, connective-tissue organization and immune modulation.	HAV may represent a musculoskeletal disorder with complex genetic architecture, potentially linked to connective-tissue fragility and immunometabolic mechanisms.
González-Elena et al. [28] (2021). Analytical cross-sectional study. Spain.	3b	To evaluate the association between shoe fit and HVA in school-aged children.	188 children	F = 90 M = 97 3 to 15 years.	Shoe length relative to foot length.	Weightbearing podoscopy; calibrated anthropometric ruler.	Significant correlations were found between higher HVA and insufficient shoe length.	Wearing shoes that are too short is associated with higher HVA in certain age groups.
Puszczalowska-Lizis et al. [29] (2022). Analytical cross-sectional study. Poland.	3b	To analyze the effect of functional shoe allowance (length and width) on foot morphology in children.	100 children	F = 50 M = 50 NA.	Functional shoe allowance (length and width).	Computerized podoscope; digital shoe measurements.	Children wearing shoes with insufficient functional allowance showed significantly higher HVA.	Poor shoe fit contributes to increased HVA in children.
O’Reilly et al. [36] (2021). Case–control study. Ireland.	3b	To evaluate the association between hallux valgus, gastrocnemius tightness, and genu valgum.	50 adults	F = 48 M = 2 43 ± 11.6 years.	Gastrocnemius tightness and genu valgum.	Silfverskiöld test; Q-angle measurement with goniometer; weightbearing radiographs for HAV staging; statistical analysis with χ^2^ and *t*-tests.	HAV was significantly associated with genu valgum and gastrocnemius tightness (*p* < 0.001).	Gastrocnemius shortening may contribute to HAV, particularly when combined with genu valgum.
Atbasi et al. [37] (2020). Case–control study. Turkey.	3b	To investigate the relationship between hallux valgus and flatfoot in adults using radiographic assessment.	213 adults	F = 0 M = 213 22.2 ± 2.8 years. 54 HAV, 159 controls.	HVA, IMA, talonavicular coverage angle, Meary’s angle, calcaneal pitch, lateral talocalcaneal angle.	Weightbearing AP and lateral radiographs; digital measurements using Pi-view Star v.5.0.7; *t*-tests and Pearson’s correlations.	HAV patients showed significantly lower calcaneal pitch and higher lateral talocalcaneal angle. HVA/IMA correlated negatively with both angles.	Flatfoot is strongly correlated with HAV, suggesting that increased pronation and reduced medial arch height contribute to HAV development.
Cruz et al. [38] (2019). Case–control study. Brazil.	3b	To determine whether HAV includes intrinsic first metatarsal rotation as a structural deformity using multiplanar CT.	91 adults	F = 69 M = 22 50.6 ± 15.8 years 146 feet (82 HAV and 64 controls).	Intrinsic 1st metatarsal rotation, DMAA, IMA, HVA, sesamoid position, round sign.	Multislice CT; multiplanar reconstruction with OsiriX MD; measurement of base-to-head rotation; mixed linear model adjusted for age and sex.	Mean 1st metatarsal rotation was 15.36° in HAV vs. 3.45° in controls. Rotation correlated with DMAA but not with HVA or IMA.	HAV involves intrinsic pronation of the 1st metatarsal as a structural deformity rather than a purely adaptive change.
Gómez Galván et al. [39] (2019). Case–control study. Spain.	3b	To evaluate the relationship between hallux pronation and HAV severity and to develop a radiographic method to quantify proximal phalanx pronation.	162 adults	F = 132 M = 30 57 ± 16 years. (132 HAV, 30 controls).	Proximal phalanx pronation, 1st metatarsal pronation, HVA, IMA, sesamoid displacement, nail–floor angle.	Weightbearing radiographs; experimental phalanx rotation model; linear regression formula; clinical nail–floor angle; Pearson’s correlations and *t*-tests.	HAV was significantly associated with increased proximal phalanx pronation, 1st metatarsal pronation, and higher nail–floor angle.	Proximal phalanx and metatarsal pronation are key factors in HAV severity. The nail–floor angle is a useful clinical indicator of hallux rotation.
Mortka et al. [40] (2018). Case–control study. Poland.	3b	To compare abdH activity in HAV and healthy feet using surface EMG.	86 adults	F = NR M = NR 40.2 ± 16.5 years. (44 HAV, 42 controls).	AbdH EMG activity in relation to HAV severity and joint mobility.	Surface EMG (KeyPoint System); Ag/AgCl electrodes; toe-spread-out test; MUAP amplitude and interference pattern; ANOVA, Mann–Whitney, Spearman, Pearson.	AbdH MUAP amplitude was significantly lower in HAV patients. No correlation with HAV severity, age, or ROM.	Early functional impairment of AbdH occurs in HAV, independent of angular deformity severity.
Tao et al. [41] (2018). Case–control study. China.	3b	To evaluate whether VDR polymorphisms are associated with HAV susceptibility.	428 adults	F = 369 M = 59 60.6 ± 13.8 years. 228 con HAV, 200 controls.	VDR SNPs (TaqI, BsmI, FokI, ApaI), flatfoot, TNF-α and inflammatory markers.	Multiplex SNaPshot^®^ genotyping; χ^2^ and logistic regression adjusted for age and sex.	Mutant alleles TaqI (C) and BsmI (A) were associated with increased HAV risk; FokI and ApaI showed no association.	VDR TaqI and BsmI polymorphisms contribute to genetic susceptibility for HAV.
Kimura et al. [42] (2017). Case–control study. Japan.	3b	To evaluate 3D mobility of the first ray in HAV vs. normal feet using WBCT.	20 adults	F = 20 M = 0 57 ± 10.2 years (10 HAV, 10 controls).	3D mobility of TN, CN, TMT, and MTP joints.	WBCT with custom loading device; 3D reconstruction using Analyze software and ICP algorithm.	HAV feet showed significantly greater 3D mobility in all first-ray joints (dorsiflexion, inversion, adduction).	HAV is associated with multiplanar first-ray hypermobility, not restricted to a single joint.
Perez Boal et al. [43] (2016). Case–control study. Spain.	3b	To assess whether 1st metatarsal and proximal phalanx geometry predicts HAV.	160 adults	F = 75 M = 85 49.8 ± 13.7 years. (79 HAV, 81 controls).	MT1 and PPH lengths, medial–lateral asymmetry (LDM–LDL).	Weightbearing radiographs; PACS; AutoCAD 2013; ANOVA, *t*-tests, ROC curves.	Proximal phalanx medial–lateral asymmetry predicted HAV with high accuracy (AUC = 0.95).	Bone morphology of MT1 and PPH is a strong anatomical predictor of HAV.
Menz et al. [44] (2016). Case–control study. United Kingdom.	3b	To examine lifelong footwear characteristics and HAV in older women.	2627 adults	F = 2627 M = 0 65.6 ± 9.9 years.	Toe-box shape, heel height, years of use.	Structured retrospective footwear questionnaire; line-drawing instrument; logistic regression.	Narrow toe-box use between ages 20–39 significantly increased HAV risk.	Early-life use of narrow shoes is associated with HAV in older age.
Nishimura et al. [30] (2014). Analytical cross-sectional. Japan.	3b	To determine HAV prevalence and associated risk factors.	1249 adults	F = 747 M = 502 58.4 ± 11.2 years.	Age, sex, BMI, footwear, joint flexibility.	Clinical exam, radiographic HVA, joint flexibility assessment.	HAV was more prevalent in women and older adults; lower BMI and reduced ankle flexibility were significant risk factors.	Aging, female sex, and joint flexibility reduction are relevant HAV risk factors.
Okuda et al. [31] (2014). Analytical cross-sectional. Japan.	3b	To determine HAV prevalence and associated factors in young Japanese women.	343 adults	F = 343 M = 0 18.7 ± 0.6 years.	Hallux pain, family history, shoe use, foot type, flatfoot, sports history, BMI, bone density.	Questionnaire; Foot Look^®^ scanner; SAI index; calcaneal densitometry.	HAV was associated with hallux pain and family history; not with footwear or flatfoot.	In young women, HAV is mainly related to hereditary factors and hallux pain.
Nery et al. [46] (2013). Case–control study. Brazil.	3b	To compare etiologic and radiographic characteristics of HAV in men vs. women.	62 adults	F = 31 M = 31 40.4 ± 16.3 years.	Family history, footwear, MPV, flatfoot.	Clinical review; weightbearing radiographs; χ^2^, *t*-tests, Mann–Whitney, Spearman.	68% of men had maternal family history; HAV was more severe in men.	HAV in men is largely hereditary, early-onset, and more severe.
Steinberg et al. [45] (2013). Case–control study. Israel.	3b	To analyze lower-limb alignment and joint laxity in older women with HAV.	49 adults	F = 49 M = 0 64 ± 10 years (25 HAV, 24 controls).	Q-angle, tibiofemoral angle, rearfoot angle, generalized hypermobility.	Weightbearing radiographs; goniometry; Beighton score.	HAV participants had greater hypermobility and altered lower-limb alignment.	Lower-limb malalignment and joint laxity contribute to HAV.
Nguyen et al. [51] (2010). Analytical cross-sectional. USA.	3b	To identify HAV risk factors in adults ≥70 years.	600 adults	F = 386 M = 214 77.9 ± 5.6 years.	Age, sex, BMI, foot pain, flatfoot, heel height.	Clinical exam; MatScan baropodometry; Poisson regression.	In women, HAV was associated with low BMI and past high-heel use; in men, with high BMI and flatfoot.	Etiologic mechanisms differ by sex.
Klein et al. [32] (2009). Analytical cross-sectional. Austria.	3b	To evaluate the relationship between shoe length and hallux angle in preschool children.	858 children	F = 439 M = 419 3 ± 6.5 years.	Interior shoe length.	3D foot scanner; shoe interior measuring device.	Insufficient shoe length increased HVA.	Small shoes are associated with lateral hallux deviation.
Munuera et al. [47] (2008). Case–control study. Spain.	3b	To assess whether MT1 and hallux length are associated with early HAV.	152 adults	F = 68 M = 84 37.3 ± 10.0 years.	Relative MT1 and hallux length.	Weightbearing radiographs; standardized measurement.	HAV cases had significantly longer MT1 and hallux.	Increased longitudinal bone length predisposes to early HAV.
Piqué-Vidal et al. [34] (2007). Descriptive cross-sectional. Spain.	4	To analyze the inheritance pattern of HAV by constructing three-generation pedigrees in patients with HAV.	350 adults	F = 328 M = 22 47.8 ± 14.8 years.	Family history, maternal/paternal lineage.	Family history questionnaire; HVA measurement; χ^2^, *t*-test, non-parametric tests.	90% had at least one affected relative; 56% penetrance; predominantly maternal transmission.	HAV follows an autosomal-dominant pattern with incomplete penetrance.
Coughlin et al. [33] (2007). Descriptive cross-sectional. USA.	4	To analyze demographics, clinical features, and family history in surgically treated HAV.	602 adults	F = 511 M = 91.	Family history, footwear, foot morphology.	Clinical and radiographic evaluation; descriptive analysis.	63% had family history; more severe deformity in women and familial cases	HAV has strong hereditary influence; footwear is not a primary cause.
Arinci Incel et al. [9] (2003). Case–control study. Turkey.	3b	To evaluate muscle imbalance between abductor and adductor hallucis in HAV.	40 adults.	F = 17 M = 3 44.5 ± 12.5 years.	EMG activity of AbdH, AddH, FHB, EHL.	Surface EMG; MUAP amplitude; interference pattern.	AbdH activity was significantly decreased in HAV.	Muscle imbalance may contribute to HAV progression.
Mancuso et al. [48] (2003). Case–control study. USA.	3b	To examine MT1 length, metatarsal head shape, IMA, and HAV development.	210 adults	F = 173 M = 37 42.1 ± 15.42 years. 110 HAV, 100 controls).	MT1 length, protrusion distance, head shape.	Weightbearing radiographs; morphological classification.	HAV was associated with long MT1 and round head; MT1 length correlated with severity.	Long MT1 is a clear risk factor for HAV.
Hardy et al. [49] 1951). Case–control study. United Kingdom.	3b	To identify anatomical and functional factors associated with HAV using radiographs, footprints, and biomechanical tests.	173 adults	F = 139 M = 34 34.3 ± 14.9 years. (89 HAV, 84 controls).	HVA, IMA, metatarsal protrusion, sesamoid displacement, hallux rotation, TMT mobility, family history.	Radiographs; photographic analysis; footprint exam; goniometry; isometric strength tests.	Strong correlation between HVA and IMA; increased hallux rotation and sesamoid displacement with severity; MT1 longer and more mobile in HAV; 63% had positive family history.	HAV involves structural deformity, increased first-ray mobility, and strong hereditary influence.

HAV: hallux abducto valgus; HVA: hallux valgus angle; IMA: intermetatarsal angle; MA: metatarsus adductus angle; PACS: Picture Archiving and Communication System; BMI: body mass index; GWAS: genome-wide association study; OR: odds ratio; DNA: deoxyribonucleic acid; PCR-RFLP: polymerase chain reaction–restriction fragment length polymorphism; TMT: tarsometatarsal; WBCT: weightbearing computed tomography; DMAA: distal metatarsal articular angle; MCA: metatarsocuneiform angle; MAA: metatarsus adductus angle; PMAA: proximal metatarsal articular angle; TN: talonavicular; CN: cuneonavicular; MTP: metatarsophalangeal; AbdH: abductor hallucis; AddH: adductor hallucis; FHB: flexor hallucis brevis; EHL: extensor hallucis longus; EMG: electromyography; MUAP: motor unit action potential; CT: computed tomography; SVM-RFE: support vector machine–recursive feature elimination; ROC: receiver operating characteristic; AUC: area under the curve.

## Data Availability

No new data were created or analyzed in this study. Data sharing is not applicable to this article.
